# Adaptation and Validation of the School Climate and School Identification Measure-Student Scale (SCASIM-St) in a Sample of Chilean Adolescents

**DOI:** 10.3389/fpsyg.2020.01561

**Published:** 2020-07-07

**Authors:** José Luis Gálvez-Nieto, Daniela Vera-Bachmann, Ítalo Trizano-Hermosilla, Karina Polanco-Levican, Claudio Briceño-Olivera

**Affiliations:** ^1^Departamento de Trabajo Social, Universidad de La Frontera, Temuco, Chile; ^2^Instituto de Psicología, Universidad Austral de Chile, Puerto Montt, Chile; ^3^Departamento de Psicología, Universidad de La Frontera, Temuco, Chile; ^4^Departamento de Psicología, Universidad Católica de Temuco, Temuco, Chile

**Keywords:** school climate, school identification, adolescence, measurement, reliability, validity

## Abstract

The school climate construct has been linked to a series of positive outcomes in adolescence; however, the few validated instruments in Spanish have a fragile theoretical base. Consequently, the aim of this study was to adapt and validate the dual School Climate and School Identification Measure-Student (SCASIM-St) scale in a sample of Chilean adolescents. First, a linguistic adaptation of the instrument was completed, followed by a cross-sectional study; 1,456 students of both sexes participated in the study (41.1% boys and 58.9% girls), with an average age of 15.76 years (SD = 1.42), representing 17 secondary schools in Chile. Three confirmatory factor models were contrasted, the one with the best fit supported the originally proposed structure. The evidence of external criterion validity, confirmed through the Authoritative School Climate Survey (ASCS), showed a significant positive relationship between the two measures. This study verified the psychometric quality for the SCASIM-St scale, allowing for its use in the Chilean context. This instrument provides a measurement tool with a solid theoretical base that can be used in the Chilean context.

## Introduction

Despite a relative consensus regarding the benefits of school climate (Steffgen et al., 2013), its definition has a wide range of conceptualizations (Alonso-Tapia and Nieto, 2019) and a lack of consistent theoretical approaches (Ramelow et al., 2015). The studies with the greatest evidence and methodological robustness (Thapa et al., 2013; Rudasill et al., 2018) agree that school climate is a complex construct that must be measured from a multidimensional perspective.

Among the currently proposed dimensions, school identification (Miller et al., 2015, 2016; Reynolds et al., 2015), attachment to the school (Finn, 1989), and connection (Loukas et al., 2006; Bizumic et al., 2009) are of great relevance since these factors have shown that schools have an intrinsic impact on their students’ behavior (Lee et al., 2017). Students who identify more strongly with their school are more likely to behave in accordance with its rules, values, and beliefs (Reynolds et al., 2015). Thus, by providing a space for building shared social identity, schools have an intrinsic impact on shaping individual behavior.

The present study focused on adolescence, a developmental stage in which individual behaviors are strongly influenced. The World Health Organization (2020) defines adolescence as the period of growth after childhood, ranging from approximately 10–19 years of age. However, some specialists in Chile use this range in a referential and flexible way, expanding the period of adolescence to account for the biological changes and the transition into social roles experienced by the youth (Gaete, 2015). Adolescence is considered a period of great growth and biological development, as well as a period in which a wide variety of risk factors can affect people’s health (World Health Organization, 2020).

### Main Psychometric Studies That Evaluate the Construct of School Climate in Chile

Despite the relevance of the school climate construct and the international evidence that justifies its study, little progress has been made in Chile in terms of the development of measurement scales and/or the adaptation of instruments. There are at least two psychometric studies that have provided evidence that the Questionnaire to Assess School Social Climate (CECSCE) is a good measurement of school climate (Gálvez-Nieto et al., 2015a; Gálvez-Nieto et al., 2017). Despite the fact that the CECSCE has shown stability in terms of its factor structure, the number of dimensions is insufficient, threatening the validity of the theoretical content of the scale.

Another scale that presents adequate levels of reliability and validity in Chile is the “School Climate Scale” (López et al., 2014). This scale consists of 18 items and has demonstrated adequate psychometric properties for a structure of four correlated factors: social support, rules, rules against violence, and participation (López et al., 2014). Although these instruments are supported by psychometric studies that endorse their relevance and use, the basic theoretical proposals lack adequate coverage and dimensional content, conflicting with the current international literature which establishes more complete multidimensional constructs (Thapa et al., 2013; Rudasill et al., 2018).

Consistent with the international literature, the measurement proposal of the dual School Climate and School Identification Measure-Student (SCASIM-St) scale is based on Bronfenbrenner’s (2002) theory of ecological systems, one of the most widely used models in the study of school climate. This theory suggests that individual behaviors are explained by the multiple social contexts in which the adolescent takes part (Rudasill et al., 2018). SCASIM-St presents a structure based on a second-order factor called School Climate, which groups five first-order factors, four of which evaluate school climate. These factors include Student–Student Relations, a factor related to the social relationships established between classmates; Student–Staff Relations, defined as the relationships between students and the administrative staff of the school; Academic Emphasis, which evaluates the degree of academic support that the teacher provides so that the student meets his academic objectives; and the Shared Values and Approach factor, which has to do with the shared vision of students regarding the objectives and rules of the school. There is also a fifth first-order factor—independent of the common second-order factor—called School Identification, which targets the degree of school identity by students toward the school (Lee et al., 2017). Based on previous research, it is hypothesized that school climate will be positively related to School Identification, as it will affect the emotions, attitudes and social relations in schools, generating effects on students’ commitment to school objectives (Goodenow and Grady, 1993).

Considering the interest and relevance of studying school climate, favorable psychometric evidence regarding the theoretical structure of the SCASIM-St and the lack of robust instruments to measure school climate, the first hypothesis is that the scores on the adapted version of the SCASIM-St will maintain a second-order structure that groups four factors, plus one independent factor, in addition to adequate levels of reliability. The second hypothesis posits that the scores on the adapted version of the SCASIM-St will present significant positive correlations with the factors on the Authoritative School Climate Scale (ASCS). Thus, the general aim of the study was to adapt and validate the SCASIM-St in a sample of Chilean adolescents.

## Methods

### Participants

The total study population was 22,469 secondary school students from the Araucanía Region of Chile. Participants were affiliated with public schools (31.1%), subsidized schools (63.3%), and private schools (5.6%) (Ministerio de Educación de Chile, 2018). The participants were selected using probability and stratified sampling, with a confidence interval of 99.7%, a variance of *p* = *q* = 0.50 and a margin of error of 3.8% (Scheaffer et al., 2000).

The sample comprised of 1,456 adolescent students in Chile, representing both sexes (41.1% boys and 58.9% girls). The age range was between 13 and 20 years, with an average age of 15.76 years (SD = 1.42). The students represented 17 secondary schools: public (453), subsidized (922), and private (81).

### Instruments

Data related to age, sex, and course (school level) were collected using a sociodemographic questionnaire.

In addition, the dual SCASIM-St scale was applied. The SCASIM-St is a dual measurement that evaluates school climate and school identification (Lee et al., 2017). It is a self-report scale that measures both constructs from 38 items, which are answered on a 5-point ordinal scale (1 = totally disagree, 5 = totally agree). The SCASIM-St has a second-order factor called School Climate that groups four first-order factors: Student–Student Relations (7 items, e.g., “Students are friendly to each other”), Student–Staff Relations (9 items, e.g., “Staff care about students”), Academic Emphasis (8 items, e.g., “Teachers challenge students to do better”), and Shared Values and Approach (8 items, e.g., “The school values and goals are well understood”). Additionally, it presents a fifth factor related to the second-order factor, called School Identification (6 items, e.g., “I feel a strong connection with this school”). The evidence of reliability and validity demonstrated adequate psychometric properties for its use in Australia.

The Authoritative School Climate Survey (ASCS) was applied as a convergent measure. This scale was originally created in the United States (Gregory et al., 2010; Konold et al., 2014; Cornell and Huang, 2016). It is a self-report scale originally composed of 15 ordinal response items (1 = never, 5 = always). It has two factors: disciplinary structure (7 items, e.g., “Students are treated fairly regardless of their race or ethnicity”), which measures the degree of impartiality of school discipline; and student support (8 items e.g., “Most teachers and other adults at this school care about all students”), which assesses the perception of the support offered by teachers and professional staff at the school. The evidence of reliability and validity in the United States (Gregory et al., 2010; Konold et al., 2014; Cornell and Huang, 2016) demonstrated adequate psychometric fit indicators in terms of factor structure and reliability.

### Procedure

For the linguistic adaptation of the instrument, the criteria established by Muñiz et al. (2013) were partially followed. First, three native English speakers, who were experts in both cultures and in the study variable, were selected. Next, they translated the instrument from English to Spanish independently. To ensure content validity, each translator presented their version of the scale to the research team, recording observations about the concepts that raised questions. Then, a joint workshop took place with the translators and research team, where an agreed-upon version of the instrument was obtained. Once the final version of the instrument was ready, it was sent to an external expert with a good command of English for another back-translation of the Spanish version into English. Later, the back-translated version was compared with the original version of the scale, and it was verified that the meaning of the items was highly consistent and that there were no substantive differences between the two versions. Finally, a pilot study was conducted that allowed the quality of the items adapted during the translation process to be assessed.

For the administration of the instruments, the school directors signed a work agreement with the research team. Informed consents were then sent to the students’ parents. Once parental authorization was provided, an informed consent was applied to the students participating in the study. Once the ethical principles of the project had been established, the measuring instruments were administered in the first hour of class.

### Data Analysis

First, measures of central tendency, dispersion, and form of each of the items on the SCASIM-St were obtained. Then, to assess the structure of the scale, three confirmatory factor models (CFA) were contrasted—one-dimensional, bifactor, and second order—using the MPLUS software v. 8.1 (Muthén and Muthén, 2017). For the implementation of the CFA, the polychoric correlations matrix was used, recommended for modeling categorical data. For the estimation of the goodness-of-fit indices, the weighted least squares means and variance adjusted (WLSMV) method was used. This method made it possible to obtain robust indices as well as appropriate estimations of the parameters and their level of error (Flora and Curran, 2004; Finney and Di Stefano, 2006). The CFA model was evaluated from the following goodness-of-fit indices: *WLSMV*χ^2^, comparative fit index (CFI), Tucker–Lewis index (TLI), and root mean square error of approximation (RMSEA). For the CFI and TLI, values equal to or greater than 0.90 are considered a reasonable fit (Schumacker and Lomax, 2016), while values over 0.95 are considered an excellent fit (Schreiber et al., 2006). For RMSEA, values below 0.08 are considered a reasonable fit (Browne and Cudeck, 1993) and values below 0.06 are considered an excellent fit (Schreiber et al., 2006). In order to estimate the reliability, the following coefficients were used: McDonald’s ω, greatest lower bound (GLB), and Cronbach’s α (Green and Yang, 2015; Trizano-Hermosilla and Alvarado, 2016).

## Results

### Descriptive Analyses

The descriptive analysis for the mean of the items yielded a maximum of 4.32 (SD = 0.846) for item 22 “The teachers want each student to do their best” and a minimum mean of 2.96 (SD = 0.996) for item 4 “The students treat each other with respect”. With respect to the measures of form, items 22 (Skewness = −1.445; Kurtosis = 2.842) and 11 “The staff treats students with respect” (Skewness = −1.288; Kurtosis = 2.016) had the highest indices of asymmetry. The results of the descriptive analysis show that the majority of students perceive the school climate as moderate, given that the average item scores are high (Table 1).

**TABLE 1:** Descriptive statistics for the items.

Items	Mean	Std. Deviation	Skewness	Kurtosis
it1	3.14	0.969	–0.347	0.023
it2	3.52	0.892	–0.536	0.446
it3	3.22	0.918	–0.362	0.155
it4	2.96	0.996	–0.225	–0.247
it5	3.00	0.940	–0.239	0.000
it6	3.20	0.936	–0.354	–0.042
it7	3.49	1.094	–0.460	–0.327
it8	4.06	0.899	–1.064	1.320
it9	3.99	0.925	–0.924	0.831
it10	4.07	0.875	–0.837	0.612
it11	4.26	0.843	–1.288	2.016
it12	3.78	0.984	–0.665	0.142
it13	3.51	1.008	–0.460	–0.027
it14	3.85	0.952	–0.787	0.545
it15	3.83	0.967	–0.728	0.327
it16	3.82	1.020	–0.754	0.220
it17	4.00	1.005	–0.995	0.705
it18	4.06	0.892	–0.885	0.694
it19	3.95	0.971	–0.871	0.525
it20	3.88	0.878	–0.746	0.824
it21	4.04	0.902	–0.841	0.511
it22	4.32	0.846	–1.445	2.482
it23	4.11	0.991	–1.145	1.037
it24	3.95	0.929	–0.864	0.746
it25	3.50	0.973	–0.427	0.100
it26	3.44	0.991	–0.454	0.111
it27	3.53	0.985	–0.531	0.187
it28	3.77	0.911	–0.484	0.006
it29	3.86	0.942	–0.714	0.368
it30	3.52	1.029	–0.468	–0.104
it31	3.93	0.976	–0.834	0.404
it32	3.81	1.019	–0.706	0.131
it33	3.64	1.123	–0.667	–0.086
it34	3.64	1.095	–0.624	–0.110
it35	3.22	1.105	–0.277	–0.421
it36	3.22	1.121	–0.285	–0.455
it37	3.49	1.128	–0.527	–0.335
it38	3.93	0.993	–0.930	0.707

### Factor Structure

**FIGURE 1:**
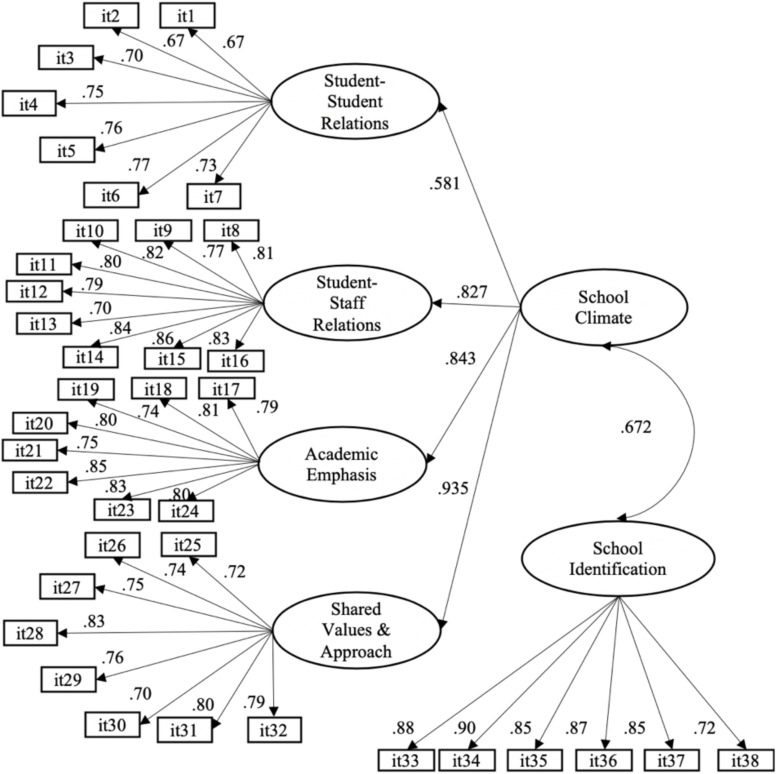
Second-order model and four factors, plus one independent factor. The factor loadings and the correlation between factors were statistically significant (*p* < 0.001).

To analyze the factor structure of the SCASIM-St, three alternative CFA models with the 38 items were considered. The first model evaluated was the one factor model, which gave an unsatisfactory fit: *WLSMV*χ^2^ (664) = 17826.295, *p* < 0.001; CFI = 0.817; TLI = 0.806; RMSEA = 0.115 (CI90% = 0.113–0.116). The second model tested was the bifactor model, which presented an acceptable fit: *WLSMV* χ*^2^* (629) = 5584.712, *p* < 0.001; CFI = 0.947; TLI = 0.941; RMSEA = 0.063 (CI90% = 0.062–0.065). Finally, the estimation of a second-order model that groups four factors plus an independent factor (Figure 1) was the one that obtained the best fit: *WLSMV*χ^2^ (660) = 4553.020, *p* < 0.001; CFI = 0.958; TLI = 0.956; RMSEA = 0.055 (CI90% = 0.053–0.056). These results indicate that the model fits the data adequately, confirming the original theoretical structure of SCASIM-St in the sample of Chilean adolescents. Figure 1 shows the confirmatory factor structure and that all the factor loadings of the items resulted in satisfactory and statistically significant values (*p* < 0.001). With respect to the four first-order factors, the highest factor loading was obtained by Shared Values and Approach (0.935). The independent factor School Identification had a moderate positive correlation with the general School Climate factor.

### Validity of External Criterion

**FIGURE 2:**
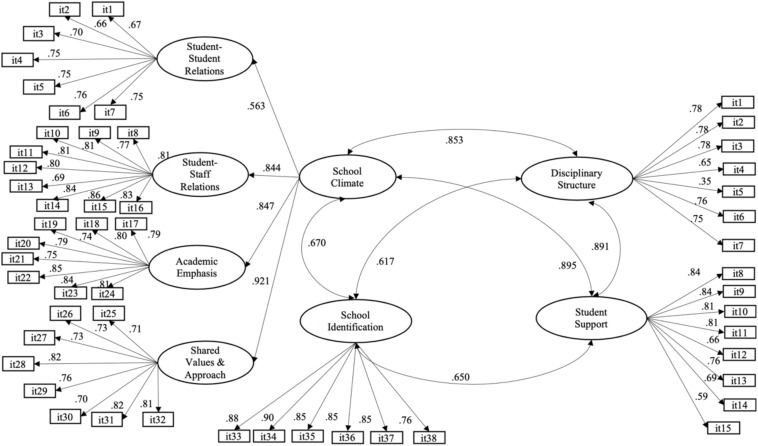
Structural model of relations between SCASIM-St and ASCS. The factor loadings and the correlation between factors were statistically significant (*p* < 0.001).

A structural equation model that correlated the factors of the SCASIM-St and ASCS was tested (Figure 2). The result showed satisfactory goodness-of-fit indices: *WLSMV* χ^2^ = 7568.305, *p* < 0.001, CFI = 0.950, TLI = 0.948, RMSEA = 0.049 (CI 90% = 0.048–0.050), presenting significant positive correlations between the second-order factor on the SCASIM-St and both factors on the ASCS, underscoring a high correlation with the factor Student Support (*r* = 0.895; *p* < 0.001). The factor Student Identification presented moderate positive relationships with these two factors. These results were consistent with the initial research hypothesis, which stated that higher scores in the School Climate and School Identification dimensions would be associated with higher scores in disciplinary structure and student support.

### Evidence of Reliability

With respect to the evidence of reliability (Table 2), all factors presented satisfactory values. The Student–Staff Relations factor demonstrated the highest reliability, while the Student–Student Relations factor showed the lowest reliability. These results indicate that the dimensions are measured accurately and that as expected, the alpha coefficient generates an underestimation of the reliability when working with congeneric data; consequently, the estimations given by McDonald’s ω and greatest lower bound coefficients are more adequate than those of Cronbach’s α. It should be noted that the SCASIM-St is a reliable scale and that each item contributes to the measurement of school climate.

**TABLE 2:** Evidence of reliability.

				95% Confidence Interval	
Dimension	McDonald’s ω	Greatest lower bound	Cronbach’s α	Lower	Upper
Student–Student Relations	0.840	0.849	0.838	0.825	0.850
Student–Staff Relations	0.916	0.921	0.915	0.908	0.921
Academic Emphasis	0.897	0.919	0.896	0.888	0.904
Shared Values Approach	0.869	0.904	0.863	0.852	0.873
School Identification	0.901	0.928	0.898	0.890	0.906

## Discussion

The aim of this study was to adapt and validate the dual SCASIM-St scale in a sample of Chilean adolescents. The results of this study show the total fulfillment of the first hypothesis, which posited that the scores of the SCASIM-St would maintain a second-order structure that groups four factors plus one independent factor, as well as adequate levels of reliability. These findings confirm the presence of a theoretical structure consistent with the Australian study (Lee et al., 2017). With respect to the fulfillment of the second hypothesis, the results demonstrate that the scores of the adapted version of the SCASIM-St present significant positive correlations with the factors on the ASCS.

The SCASIM-St provides a measurement of school climate based on Bronfenbrenner’s (2002) theory of ecological systems. Ecological systems theory provides robust theoretical support to the scale’s factor structure, an aspect that has advantages over previous measures used in the Chilean context (López et al., 2014; Gálvez-Nieto et al., 2015a, 2017). The results of this study show that the SCASIM-St offers a novel measurement scale for future studies in Spanish, in addition to providing a tool that will contribute valid and reliable data to assist in school decision-making.

The theoretical and conceptual implications of this study show that Bronfenbrenner’s (2002) theory of ecological systems is an adequate framework to support the concept of school climate, as it highlights the multidimensionality of the construct (Rudasill et al., 2018). The five factors that make up this construct provide a variety of environmental elements that can contribute to the development of the school climate and provide a conceptual guide to improving educational institutions, both academically and organizationally.

The findings of this investigation should be interpreted with caution due to the following limitations: First, these results were derived from a cross-sectional design, but longitudinal studies are required to provide test–retest estimations of reliability and validity. Second, the study was based only on self-reported measures by students. We suggest that future studies consider including instruments that measure school climate from the perspective of teachers or parents in addition to students. Third, the sample represents a region of Chile which may be similar to other areas of the country, but the results cannot be generalized to the entire country.

Future lines of enquiry could assess associations with other constructs linked to the school setting. In this context, it would be interesting to see the effect of school climate on school regulations (Gálvez-Nieto et al., 2015b) or explanatory studies that analyze the relationships between the various ecological aspects of the school system (Bronfenbrenner, 2002; Gálvez-Nieto et al., 2020). This study highlights the need to carry out psychometric studies in order to estimate the stability of school climate measurements over time and to analyze the invariability of the construct in other populations of Spanish-speaking students, with the aim of facilitating the comparison of results between different cultural contexts.

## Data Availability Statement

The datasets generated for this study are available on request to the corresponding author.

## Ethics Statement

The studies involving human participants were reviewed and approved by the Comité de Ética de Investigación de la, Universidad de La Frontera, Temuco, Chile. Written informed consent to participate in this study was provided by the participants’ legal guardian/next of kin.

## Author Contributions

JG-N created the research question, conducted bibliographic search, methodological design, contributed to analysis, results, and discussion. DV-B conducted bibliographic search, theoretical framework, integrated results, and contributed to the discussion. ÍT-H contributed to the methodological design, performed the data analysis, and generated the results. KP-L conducted bibliographic search, theoretical framework, and contributed to the discussion. CB-O performed the data collection, contributed to analysis, results and discussion.

## Conflict of Interest

The authors declare that the research was conducted in the absence of any commercial or financial relationships that could be construed as a potential conflict of interest.
